# Regulatory Mechanisms of Sterol Regulatory Element-Binding Protein-Dependent Human ELOVL6 Expression in Response to Free Fatty Acids

**DOI:** 10.7759/cureus.104449

**Published:** 2026-02-28

**Authors:** Michiko Tajiri-Mori, Riko Kitazawa, Kiyoshi Mori, Ryuma Haraguchi, Sohei Kitazawa

**Affiliations:** 1 Department of Pathology, Kobe University Graduate School of Medicine, Kobe, JPN; 2 Department of Diagnostic Pathology, Ehime University Hospital, Toon, JPN; 3 Department of Central Laboratory and Surgical Pathology, Osaka National Hospital, National Hospital Organization, Osaka, JPN; 4 Department of Molecular Pathology, Ehime University Graduate School of Medicine, Toon, JPN

**Keywords:** elvol6, human, promoter, sre, srebp

## Abstract

*ELOVL6* is a key microsomal enzyme that catalyzes the elongation of C16 saturated and monounsaturated fatty acids into C18 species and plays a pivotal role in lipid homeostasis. Although *ELOVL6* is recognized as a downstream target of sterol regulatory element-binding proteins (SREBPs), the promoter architecture and the functional contribution of individual sterol regulatory elements (SREs) within the human *ELOVL6* gene remain poorly characterized. In this study, we investigated the transcriptional regulation of human *ELOVL6* in response to free fatty acids, focusing on SREBP-mediated mechanisms. A 1.6 kb genomic region encompassing the human *ELOVL6* promoter and first intron was cloned, and two putative SREs, designated SRE1 and SRE2, were identified. Using human hepatoma Huh7 cells, we found that palmitic acid (PA), but not oleic acid (OA), induced a transient upregulation of *ELOVL6* mRNA expression at 24 h, which diminished by 48 h. Electrophoretic mobility shift assays and chromatin immunoprecipitation analyses demonstrated specific binding of SREBP to both SRE1 and SRE2, with enhanced recruitment following PA stimulation. Luciferase reporter assays revealed that site-directed mutagenesis of either SRE significantly reduced basal promoter activity, while simultaneous disruption of both elements resulted in profound suppression, indicating that both elements are essential for fatty acid-responsive transcription. Consistent with these findings, PA stimulation caused a rapid but transient accumulation of nuclear SREBP, peaking at 4 h and declining thereafter, suggesting that the short nuclear half-life of active SREBP contributes to the transient induction of *ELOVL6*. Collectively, these results delineate the cis-regulatory framework of the human *ELOVL6* gene and demonstrate that SRE1 and SRE2 cooperatively mediate SREBP-dependent, fatty acid-responsive transcription. This tightly regulated and transient activation of* ELOVL6 *may represent an adaptive mechanism to acute lipid-derived stress, the dysregulation of which could contribute to metabolic disorders and cancer-associated lipid remodeling.

## Introduction

*ELOVL6* is a microsomal enzyme belonging to the* ELOVL *family that catalyzes the elongation of C16 saturated and monounsaturated fatty acids to C18 species [1]. By modulating intracellular fatty acid composition, *ELOVL6* plays a fundamental role in lipid biosynthesis and contributes to maintaining membrane properties and metabolic homeostasis [1]. In metabolic tissues such as the liver and adipose tissue, *ELOVL6* expression is dynamically regulated in response to nutritional and hormonal stimuli, indicating a critical role in energy metabolism [2,3].

The physiological significance of *ELOVL6* has been demonstrated by studies using *ELOVL6*-deficient mice [1]. *ELOVL6* knockout mice develop obesity and hepatic steatosis comparable to wild-type (WT) mice when fed a high-fat diet while maintaining preserved insulin sensitivity and normoglycemia [4]. These findings suggest that qualitative alterations in hepatic fatty acid composition, rather than lipid accumulation per se, critically influence glucose homeostasis [5]. In line with this concept, dysregulated *ELOVL6* expression has been implicated in metabolic disorders such as insulin resistance [1,6-8] and metabolic dysfunction-associated steatotic liver disease (MASLD; formerly non-alcoholic fatty liver disease (NAFLD)) [9,10].

At the transcriptional level, *ELOVL6* expression is regulated by lipogenic transcription factors, including sterol regulatory element-binding proteins (SREBPs), which coordinate fatty acid and cholesterol metabolism in response to cellular lipid status [11]. Although *ELOVL6* is recognized as a downstream target of SREBP signaling, the promoter architecture of the human *ELOVL6* gene and the functional contribution of individual SREBP-responsive cis-regulatory elements remain poorly defined [11]. In particular, how extracellular metabolic cues, such as free fatty acids (FFAs), are integrated at the human *ELOVL6* promoter level has not been fully elucidated.

In this study, we hypothesized that putative SRE1 and SRE2 sequences contribute to SREBP-mediated transcriptional regulation of the human *ELOVL6* gene in response to FFAs. We sought to define the cis-regulatory framework underlying SREBP-dependent transcriptional control of the human *ELOVL6* gene. We cloned a 1.6 kb genomic region encompassing the promoter and first intron and identified two putative sterol regulatory elements (SRE1 and SRE2). Using electrophoretic mobility shift assays (EMSAs), chromatin immunoprecipitation (ChIP), site-directed mutagenesis, luciferase (Luc) reporter assays, and temporal analysis of nuclear SREBP accumulation, we tested the hypothesis that these SREs cooperatively mediate fatty acid-responsive transcription of human *ELOVL6*. Through this approach, we aimed to clarify the molecular mechanism linking lipid-derived signals to the transcriptional regulation of fatty acid elongation.

## Materials and methods

Cell culture

The human hepatoma cell line Huh7 was maintained in DMEM (low glucose) supplemented with 10% fetal bovine serum and antibiotics at 37°C in a humidified atmosphere containing 5% CO₂. Palmitic acid (PA) and oleic acid (OA) were dissolved in 0.1 N NaOH at 70°C and conjugated to fatty acid-free bovine serum albumin (BSA) at a 6:1 molar ratio. The final fatty acid concentration was 500 μM. Control cells received BSA vehicle alone at the same final concentration.

The human hepatoma cell line Huh7, originally derived from a well-differentiated hepatocellular carcinoma, was obtained from the Japanese Collection of Research Bioresources (JCRB) Cell Bank (Osaka, Japan) and used for gene expression and promoter activity analyses. Cells were cultured in Dulbecco’s Modified Eagle’s Medium (DMEM) containing low glucose and supplemented with 10% fetal bovine serum under standard humidified conditions at 37°C with 5% CO₂. For fatty acid stimulation experiments, cells were treated with 500 μM OA or PA (Sigma-Aldrich, St. Louis, MO, USA) for 24 h prior to subsequent promoter analyses, reporter assays, and gene expression studies. PA and OA were dissolved in 0.1 N NaOH at 70°C and conjugated to fatty acid-free BSA at a 6:1 molar ratio. Control cells received BSA vehicle alone at the same final concentration.

Quantitative real-time polymerase chain reaction

Total RNA was isolated from cells, and complementary DNA was synthesized using standard procedures. Reverse transcription-quantitative polymerase chain reaction (RT-qPCR) was performed using TaqMan® Gene Expression Assays (Applied Biosystems). The following assay was used for human* ELOVL6*: Hs00203685_m1. Glyceraldehyde-3-phosphate dehydrogenase (GAPDH) (Hs00266705_g1) was used as an internal control. Gene expression levels were quantified using the comparative Ct method.

Electrophoretic mobility shift assay

Nuclear extracts were prepared using a commercial nuclear extraction kit. Binding reactions were performed using 5 μg of nuclear protein in binding buffer containing Tris-HCl, KCl, MgCl₂, DTT, glycerol, and poly(dI-dC). Biotin-labeled double-stranded oligonucleotides corresponding to the consensus SRE sequence (GATCCTGATCACCCCACTGAGGAG) and the putative SRE sites were synthesized. The sequences used were as follows: SRE1: ATTTCTCAAATCGCACGAGGGGGAGGAGA and SRE2: CCTCAGGTGATCCGCCCACCTCGGCCTCC.

For EMSA, biotin-labeled double-stranded oligonucleotide probes were used at a final concentration of 10-20 fmol per reaction. Nuclear extracts (5-10 μg) were incubated with probes in binding buffer for 20 minutes at room temperature. Complexes were resolved on a 6% non-denaturing polyacrylamide gel in 0.5× Tris-borate-EDTA (TBE) buffer at 100 V for approximately 1.5 h and transferred to a nylon membrane according to the manufacturer’s protocol. Nuclear extracts were prepared and incubated with labeled probes prior to electrophoresis.

Chromatin immunoprecipitation assay

ChIP assays were performed to examine in vivo binding of SREBP to the *ELOVL6 *gene. Cells were crosslinked with 1% formaldehyde for 10 min at room temperature and quenched with glycine. Chromatin was sonicated to obtain DNA fragments averaging 200-500 bp. Immunoprecipitation was performed using anti-SREBP-1 (E9F4O) rabbit monoclonal antibody (Cell Signaling Technology) or normal IgG as a negative control, followed by Protein A sepharose CL-4B (Cytiva) incubation. After rinsing, immunoprecipitated DNA was analyzed by PCR using primers flanking the SRE1 and SRE2 regions. The primer sequences were as follows: SRE1-sense: TTTCGCACACTCCCTCGCCAAGG, SRE1-antisense: CGTTTGTCCATCACCCTTTTTACTC, SRE2-sense: ACGGAGTTTCACCATGTTGGCCAGG, and SRE2-antisense: GCGGTGGTGGCTCACAGAAGTAATC.

Plasmid construction and mutagenesis

A 1.6 kb genomic fragment of the human *ELOVL6* gene was cloned into Luc reporter vectors. Initial cloning was performed using the TA Cloning® Dual Promoter kit (Invitrogen), followed by subcloning into pGL4 Luc reporter vectors (Promega). Site-directed mutagenesis of the putative SREs (SRE1: TCGCACGAG to CCACATCGC, and SRE2: ATCCGCCCAC to ATCCTTTTAC) within the ELOVL6 first intron and promoter was carried out using the QuikChange® II XL Site-Directed Mutagenesis Kit (Stratagene, Agilent Technologies, Santa Clara, CA, USA), according to the manufacturer’s instructions. All constructs were verified by DNA sequencing.

Luciferase reporter assay

Cells were seeded in 24-well plates at a density of 2×10⁵ cells per well. Cells were transiently transfected with WT or mutant *ELOVL6* promoter-Luc constructs. Transfections were performed using Lipofectamine 3000 according to the manufacturer’s protocol. A total of 500 ng of Luc reporter plasmid and 20 ng of Renilla control plasmid were used per well. Following transfection, Huh7 cells were stimulated with OA or PA at a final concentration of 500 μM for 24 h. Luc activity was measured using the Dual-Luciferase® Reporter Assay System (Promega, Madison, WI, USA). Transfection efficiency was monitored by co-transfection with a Renilla Luc control plasmid and showed minimal inter-experimental variability (<10% coefficient of variation). Site-directed mutations were confirmed by direct Sanger sequencing of the entire insert, and no unintended mutations were detected.

Nuclear protein extraction and Western blot analysis

Nuclear and cytoplasmic protein fractions were prepared using the NE-PER® Nuclear and Cytoplasmic Extraction Reagents (Pierce Biotechnology, Thermo Fisher Scientific, Rockford, IL, USA). Protein concentrations were determined prior to electrophoresis, and equal amounts of protein were loaded onto each lane. Uniform protein loading was confirmed by Coomassie Brilliant Blue (CBB) staining of the gel. Protein expression was analyzed by Western blotting using a rabbit polyclonal antibody against SREBP-1 (Abcam, Cambridge, UK; cat. no. ab191857). Lamin B1 (Cell Signaling Technology, Danvers, MA, USA; cat. no. 13435) was used as a nuclear loading control. Band intensities were quantified using ImageJ software (National Institutes of Health, Bethesda, MD, USA; version 1.54f), and relative protein levels were normalized to Lamin B1 and expressed relative to control.

Statistical analysis and data presentation

All experiments were performed using three independent biological replicates, each conducted on separate days with independently prepared cell cultures and fatty acid solutions. Within each biological replicate, measurements were performed in triplicate as technical replicates. Data are presented as mean ± SD of the biological replicates. Statistical analyses were performed using Student’s t-test, and differences were considered statistically significant at P<0.05 (*) and P<0.01 (**).

## Results

Differential effects of saturated and monounsaturated fatty acids on *ELOVL6* in Huh7 cells

**Figure 1:**
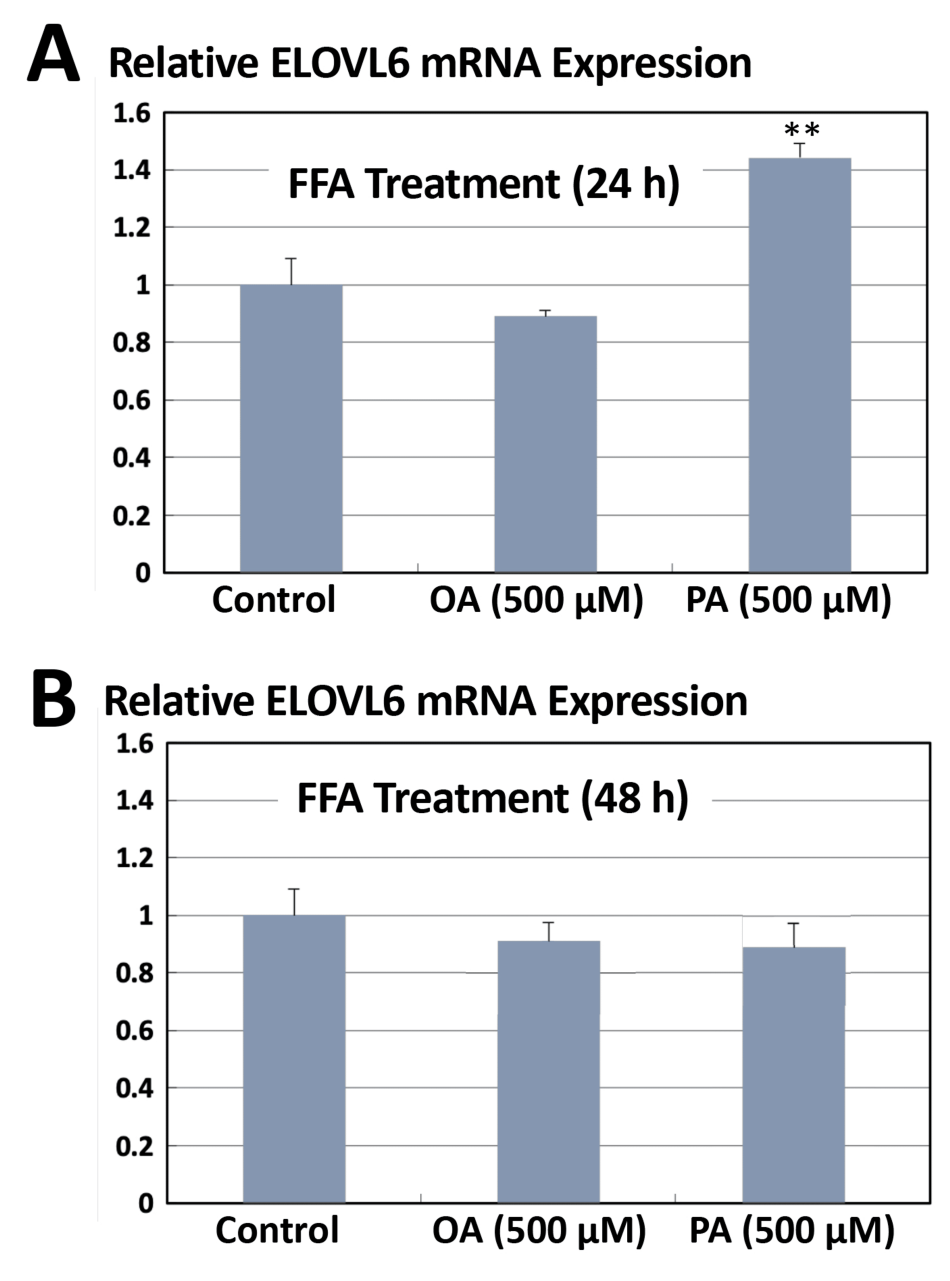
Fatty acid-dependent regulation of ELOVL6 mRNA expression in Huh7 cells ^**^Statistically significant difference (P<0.01). Huh7 cells were cultured in low-glucose conditions and treated with OA or PA. Total RNA was extracted at the indicated time points, and* ELOVL6* mRNA levels were quantified by real-time PCR using GAPDH as an internal control. Data are presented as relative expression levels normalized to untreated controls. OA: oleic acid; PA: palmitic acid; GAPDH: glyceraldehyde-3-phosphate dehydrogenase; PCR: polymerase chain reaction; FFA: free fatty acid

Huh7 human hepatocellular carcinoma cells were cultured in low-glucose DMEM and treated with 500 μM OA or PA for 24 or 48 h. The mRNA expression level of *ELOVL6* was subsequently analyzed by quantitative real-time PCR. After 24 h of treatment (Figure 1A), OA exposure did not significantly alter *ELOVL6* expression compared with untreated control cells. In contrast, PA treatment significantly upregulated *ELOVL6* mRNA levels, resulting in an approximately 1.4-fold increase relative to control (P<0.01). However, after 48 h of treatment (Figure 1B), no significant differences in *ELOVL6* expression were observed in either the OA- or PA-treated groups compared with control cells. These findings indicate that PA, but not OA, transiently induces *ELOVL6* expression in Huh7 cells, and this effect diminishes with prolonged exposure.

Genomic organization and sequence of the human *ELOVL6* promoter-exon 2 region with putative sterol regulatory elements

**Figure 2:**
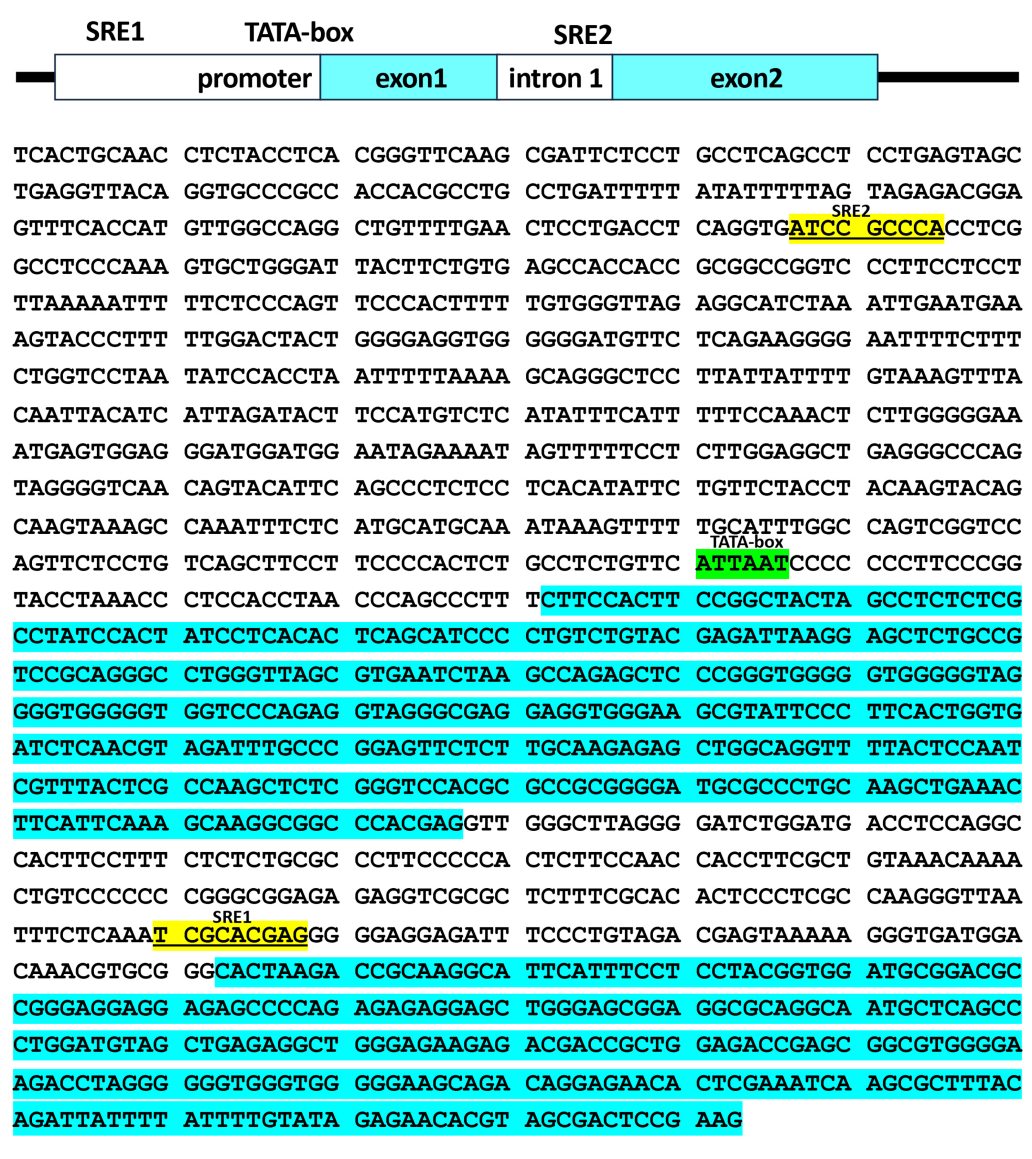
Genomic organization of the human ELOVL6 gene spanning the promoter region and first intron Schematic representation of the human *ELOVL6* genomic region analyzed in this study. The cloned fragment spans the promoter region and the first intron. Putative SRE1 and SRE2 are indicated, along with the TSS, TATA box, and exon-intron structure. TSS: transcription start site; SRE: sterol regulatory element

In this study, a ~1.3 kb genomic region of the human *ELOVL6* gene was analyzed, spanning from 750 bp upstream of the transcription start site (TSS) to a region proximal to the translation initiation site of exon 2. As illustrated in Figure 2, two putative SREs were identified within this region: one located in the first intron (SRE1) and the other in the promoter region (SRE2). The SRE sequences are underlined and highlighted in yellow. A putative TATA box is shown in green, and exon sequences are indicated in blue.

DNA-protein interaction analysis by electrophoretic mobility shift assays and chromatin immunoprecipitation assays

**Figure 3:**
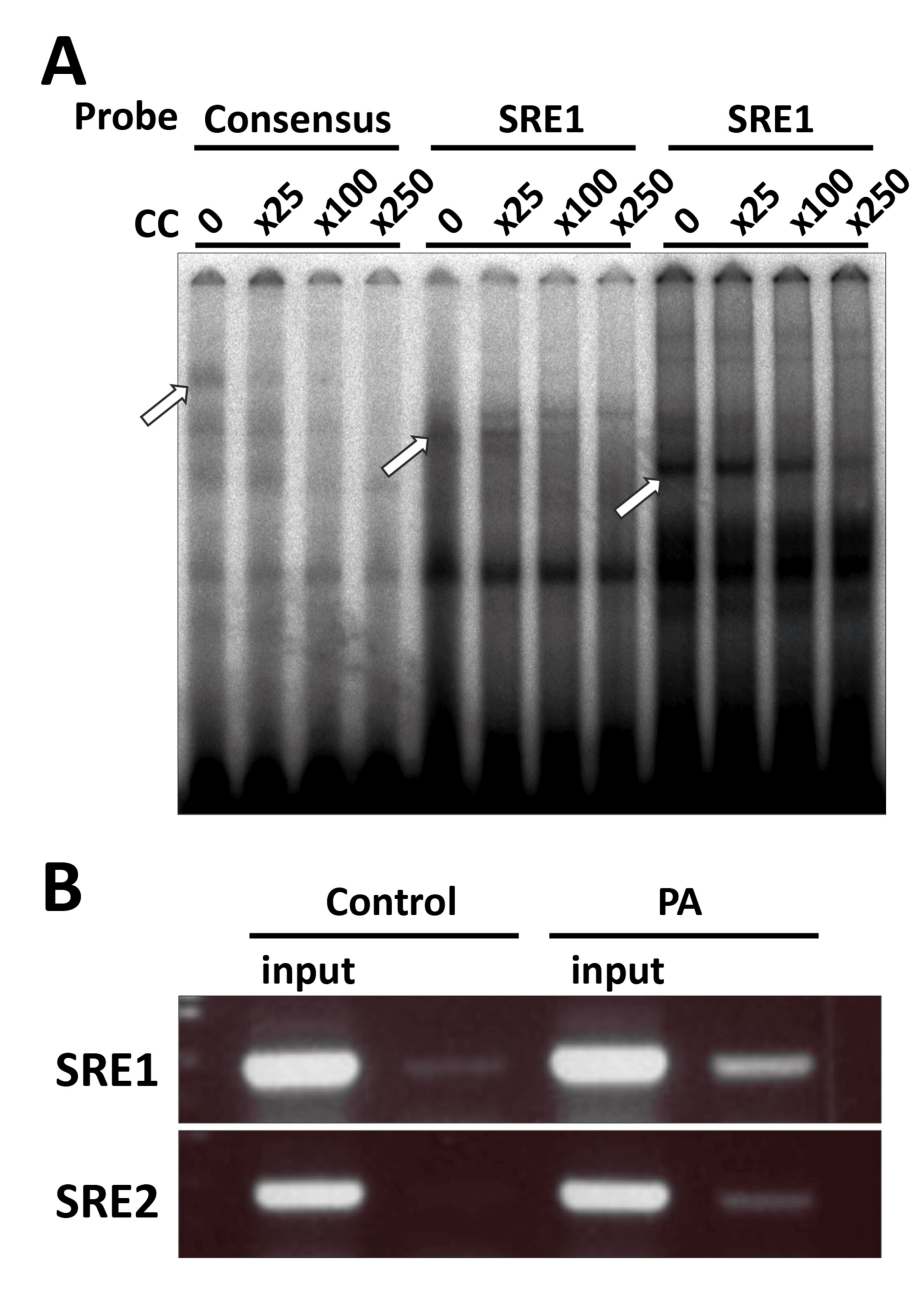
SREBP binding to putative SREs within the human ELOVL6 gene (A) EMSAs demonstrating protein-DNA complex formation using probes corresponding to the SRE consensus sequence, SRE1, and SRE2. Competition assays were performed with increasing concentrations of unlabeled oligonucleotides. (B) ChIP analysis showing in vivo association of SREBP with the SRE1 and SRE2 regions in Huh7 cells following fatty acid stimulation. SREBP: sterol regulatory element-binding proteins; CC: control condition; SRE: sterol regulatory element; EMSA: electrophoretic mobility shift assay; ChIP: chromatin immunoprecipitation

The DNA-binding activity of SREBP to the SRE consensus sequence, as well as to the SRE1 and SRE2 sequences, was examined in vitro by EMSA. As shown in Figure 3A, the DNA-protein complex formed with the SRE consensus probe was progressively diminished in a concentration-dependent manner upon addition of unlabeled (cold) competitor oligonucleotides, leading to the disappearance of the specific shifted band indicated by an open arrow. Similarly, DNA-protein complexes formed with the SRE1 and SRE2 probes were effectively competed away in a dose-dependent manner by the corresponding cold competitors, as indicated by open arrows. Figure 3B shows the results of ChIP assays performed using an anti-SREBP-1 antibody. Under control culture conditions, faint PCR-amplified bands corresponding to the SRE1 and SRE2 regions were detected. Upon treatment with PA, the intensity of these bands was markedly increased, indicating enhanced recruitment of SREBP to both SRE1 and SRE2 in vivo.

Effects of sterol regulatory element 1 and sterol regulatory element 2 mutagenesis on luciferase activity

**Figure 4:**
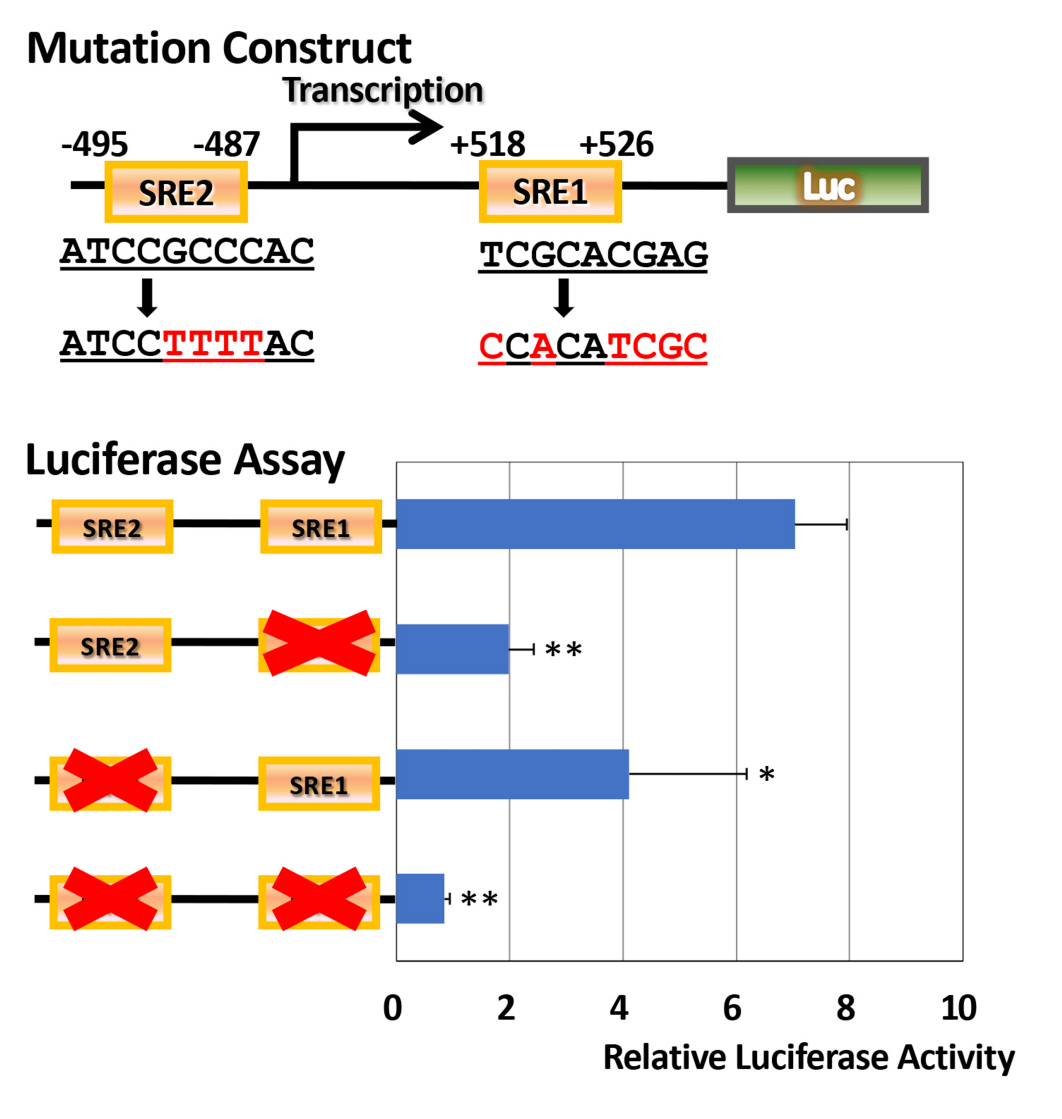
Functional contribution of SRE1 and SRE2 to ELOVL6 promoter activity ^*^Statistically significant difference, P<0.05. ^**^Statistically significant difference, P<0.01. Luc reporter constructs containing the WT *ELOVL6* promoter region spanning the promoter and first intron, or site-directed mutations in SRE1, SRE2, or both sites, were transiently transfected into Huh7 cells. Promoter activity was assessed using a dual-Luc assay and normalized to Renilla Luc activity. SRE: sterol regulatory element; Luc: luciferase; WT: wild-type

As shown in Figure 4, the WT 1.3 kb *ELOVL6* promoter construct exhibited robust Luc activity (n=3). Introduction of a site-directed mutation into SRE1 resulted in a marked and statistically significant reduction in Luc activity compared with the WT construct (two-tailed Student’s t-test, P<0.01). Mutation of SRE2 also significantly decreased promoter activity (n=3), corresponding to an approximately 50% reduction relative to the WT construct (P<0.05). Notably, simultaneous disruption of both SRE1 and SRE2 led to a profound suppression of Luc activity (n=3), which was significantly lower than that of the WT construct (P<0.01).

Differential responsiveness of *ELOVL6* promoter constructs to oleic acid and palmitic acid stimulation

**Figure 5:**
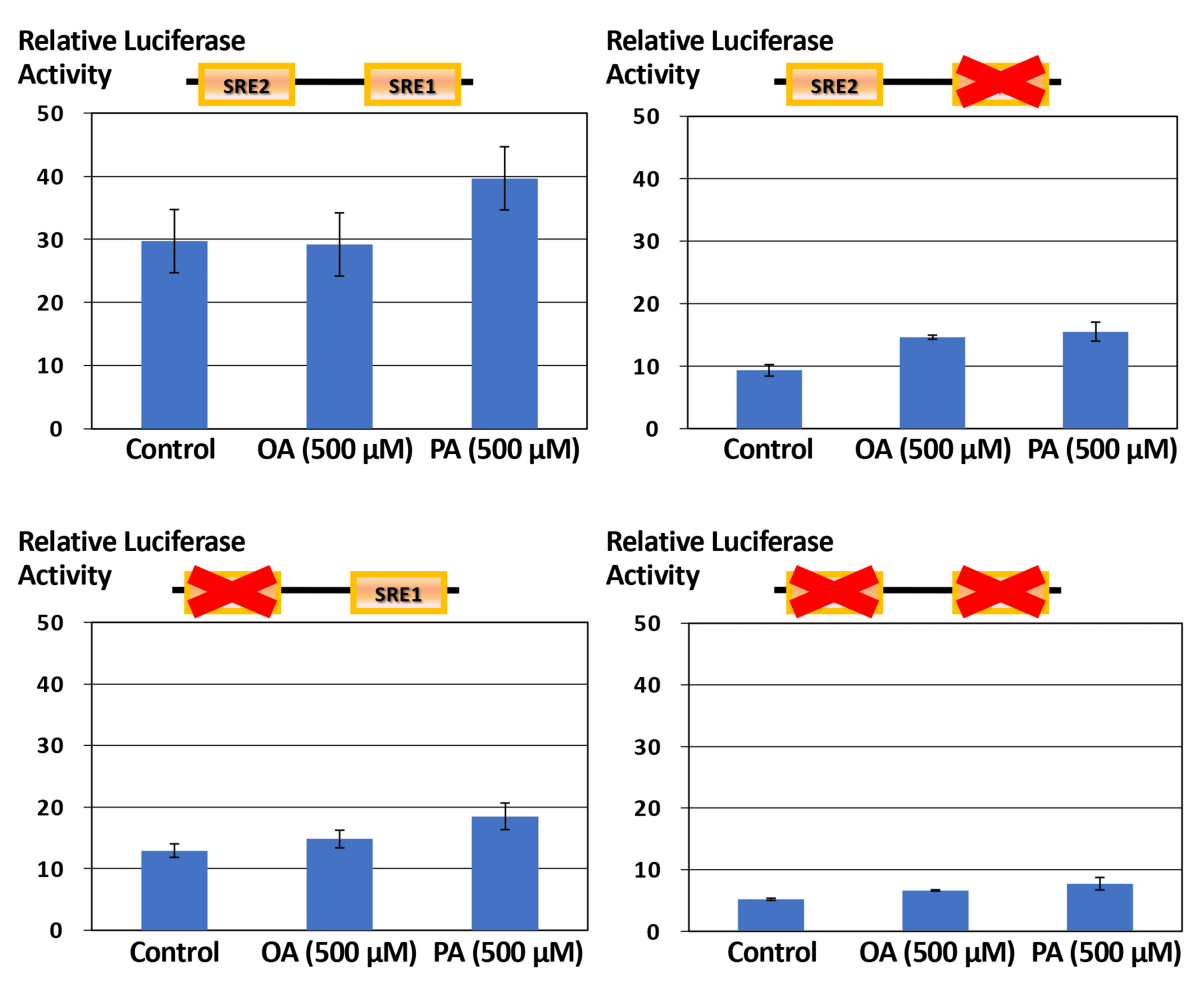
Fatty acid responsiveness of WT and mutant ELOVL6 gene constructs spanning the promoter and first intron Huh7 cells transfected with WT or SRE-mutant *ELOVL6* gene spanning the promoter and first intron-Luc constructs were treated with OA or PA. Relative Luc activities were compared to evaluate the requirement of intact SREs for fatty acid-dependent transcriptional regulation. OA: oleic acid; PA: palmitic acid; SRE: sterol regulatory element; WT: wild-type; Luc: luciferase

Figure 5 illustrates the responsiveness of each *ELOVL6* promoter construct to OA and PA. In the WT construct, OA treatment did not result in a significant change in Luc activity compared with the control condition (mean±SD, n=3, Student’s t-test, P>0.05), whereas PA stimulation induced an approximately 1.3-fold increase in promoter activity. However, this increase did not reach statistical significance under the present experimental conditions (mean±SD, n=3, Student’s t-test, p>0.05). This modest response was directionally consistent with the real-time PCR results shown in Figure 1. In contrast, in constructs harboring mutations in either SRE1 or SRE2, the stimulatory effects of both OA and PA on Luc activity were markedly attenuated, indicating that intact SRE1 and SRE2 are required for fatty acid-responsive transcriptional activation of the *ELOVL6* promoter.

Time-dependent nuclear sterol regulatory element-binding protein changes after palmitic acid treatment by Western blot

**Figure 6:**
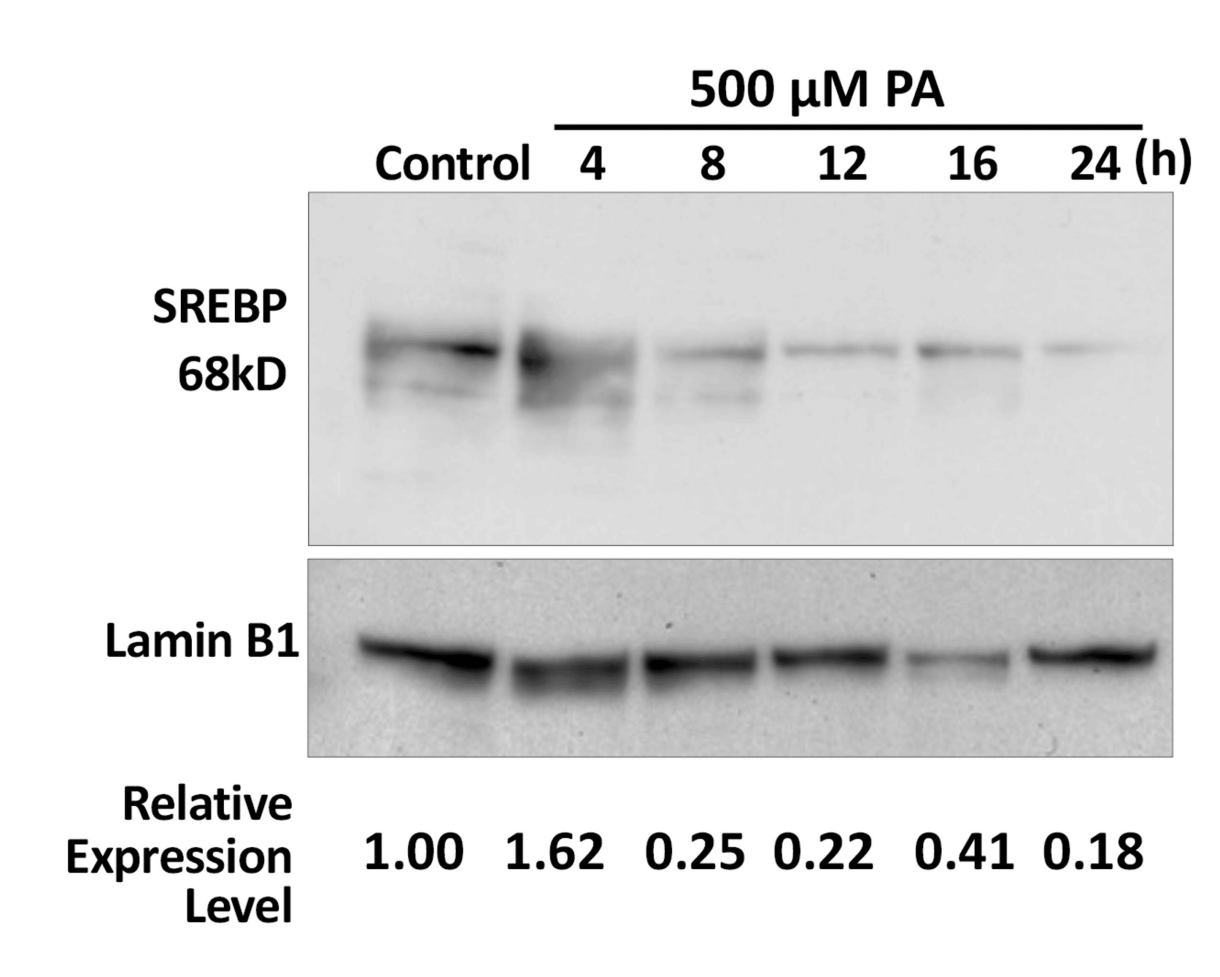
Time-dependent changes in nuclear SREBP levels following PA treatment Nuclear extracts were prepared from Huh7 cells at the indicated time points after PA stimulation. SREBP protein levels were analyzed by Western blotting. Lamin B1 was used as a nuclear loading control. Band intensities were quantified and normalized to Lamin B1, and values were expressed relative to control conditions. SREBP: sterol regulatory element-binding protein; PA: palmitic acid

As shown in Figure 6, nuclear SREBP was detectable under basal control conditions. Following PA treatment, nuclear SREBP levels increased and reached a peak of approximately 1.4-fold relative to control at 4 h. Thereafter, nuclear SREBP levels gradually declined (0.8-fold at 8 h, 0.78-fold at 12 h, 0.85-fold at 16 h, and 0.62-fold at 24 h), returning to control levels or below at later time points. This temporal pattern indicates a transient nuclear accumulation of SREBP in response to PA stimulation.

## Discussion

In this study, we characterized the cis-regulatory architecture of the human *ELOVL6* gene and demonstrated that two SREs located in the promoter region and first intron cooperatively mediate SREBP-dependent transcriptional activation in response to saturated fatty acids. Our findings provide direct functional evidence that human *ELOVL6* expression is tightly controlled at the transcriptional level through specific SREBP-SRE interactions. This study was conducted using the Huh7 hepatoma cell line, a well-established model of hepatic lipid metabolism. Although this system allows controlled mechanistic investigation, validation in primary hepatocytes or in vivo models will be necessary to determine the broader physiological relevance of these findings. The concentration of PA (500 μM) used in this study was selected based on commonly employed in vitro models of lipid-induced SREBP activation. However, this concentration may exceed typical physiological plasma levels and could partially reflect lipotoxic stress. Future studies using dose-response analyses across a wider concentration range would help clarify this issue.

Previous studies have established *ELOVL6* as an important regulator of lipid metabolic balance and have linked its dysregulation to metabolic disorders such as MASLD [1,8-12]. However, the mechanisms by which lipid-derived extracellular signals are translated into transcriptional responses at the human *ELOVL6* locus have remained poorly understood. The present results address this gap by delineating the promoter elements required for fatty acid-responsive transcription and demonstrating their functional relevance in a human hepatocellular context.

Dysregulated *ELOVL6* expression has been implicated in a broad spectrum of metabolic disorders [13]. In the liver, overactivation of the SREBP1c-*ELOVL6* axis is a well-recognized feature of MASLD [2]. Moreover, mice lacking *ELOVL6* display improved insulin sensitivity and reduced diet-induced steatosis, underscoring the functional importance of this enzyme in the pathogenesis of metabolic dysfunction [4]. In humans, an increased frequency of variants in the *ELOVL6* gene has also been reported in patients with Crohn’s disease [14]. Collectively, these observations underscore the need for tight transcriptional regulation of *ELOVL6* to maintain hepatic and systemic metabolic integrity. The current findings, however, should be interpreted within the context of in vitro promoter regulation and do not provide direct evidence for disease causality, although they suggest that altered *ELOVL6* function may contribute not only to metabolic but also to inflammatory phenotypes.

Beyond its metabolic functions, aberrant upregulation of *ELOVL6* has recently gained attention in the context of cancer biology [15,16]. Elevated *ELOVL6* expression has been reported in hepatocellular carcinoma, breast cancer, colorectal cancer, and several other malignancies, where it is associated with enhanced cellular proliferation, invasive potential, and metabolic reprogramming [15-19]. The tumor-promoting effects of *ELOVL6* are thought to involve alterations in fatty acid composition, increased tolerance to endoplasmic reticulum stress, and activation of oncogenic signaling pathways [19]. These findings suggest that dysregulated *ELOVL6* expression may represent a convergent mechanism linking lipid metabolic remodeling with tumor progression.

A precise understanding of the promoter architecture and regulatory logic of *ELOVL6* is therefore essential for interpreting how metabolic and pathophysiological cues influence fatty acid elongation pathways. From this perspective, the present findings contribute to clarifying the transcriptional framework of the human *ELOVL6* gene, including features of its core promoter region, the involvement of specific cis-regulatory elements, and the transcription factors that mediate context-dependent activation. Such regulatory characteristics provide a basis for understanding how lipid-related signals are selectively integrated at the *ELOVL6* locus. Promoter structures of *ELOVL6* have been examined in several non-human species, including goat, Scylla paramamosain, Pekin duck, Larimichthys crocea, cattle, mouse, and pig [2,11,20-25]. However, detailed analyses of the human *ELOVL6* promoter have remained limited. In this study, SREs were identified within the human *ELOVL6* promoter, and notably, corresponding SREs have also been reported in these other species. This cross-species conservation supports the notion that SRE-mediated transcriptional regulation represents a fundamental and evolutionarily conserved mechanism governing *ELOVL6* expression. By contrast, promoter analyses of the human ELOVL5 gene have revealed a distinct regulatory architecture [26]. Although two SREs have also been identified in the human ELOVL5 promoter, their genomic locations differ markedly from those observed in *ELOVL6 *[26]. Specifically, the two SREs in ELOVL5 are located approximately 10 kb upstream of the TSS and within exon 1, respectively, a configuration that contrasts sharply with the proximal promoter arrangement identified for *ELOVL6* in the present study [26]. These differences highlight gene-specific variations in SRE-dependent transcriptional control among members of the ELOVL family, despite their shared roles in fatty acid elongation.

While *ELOVL6* expression is switched on and off via SRE-dependent transcriptional control, the persistence of its expression appears to be critically dependent on the duration of nuclear SREBP activity. As shown in Figure 1B, the transient induction of *ELOVL6* expression, followed by its decline within 48 h, likely reflects a tightly regulated adaptive response to acute lipid-derived stress. This temporal expression pattern can be reasonably attributed to the rapid clearance of nuclear SREBP, as demonstrated in Figure 6, where nuclear SREBP levels peak and subsequently decline within a remarkably short time frame of approximately 4 h. SREBP (125 kDa) is synthesized as an inactive precursor containing two transmembrane domains and is initially localized to the endoplasmic reticulum membrane. Upon activation, the mature form of SREBP (68 kDa) forms homodimers and translocates to the nucleus, where it functions as a transcriptional regulator. Importantly, the nuclear half-life of active SREBP is relatively short, estimated to be approximately 3 h, during which it undergoes post-translational modifications such as ubiquitination and SUMOylation, ultimately leading to proteasomal degradation [27]. The rapid decline in nuclear SREBP levels likely reflects transient nuclear accumulation followed by regulatory clearance mechanisms. However, direct degradation kinetics were not assessed in the present study. Such tight temporal control may permit short-term remodeling of fatty acid composition while preventing sustained transcriptional activation that could otherwise exacerbate endoplasmic reticulum stress and metabolic imbalance. In this physiological context, *ELOVL6* appears to function as an early-response effector that fine-tunes lipid composition in response to acute stimuli, without committing cells to long-term metabolic reprogramming.

In contrast, this finely tuned regulatory mechanism may become altered under chronic pathological conditions such as MASLD and cancer. In MASLD, persistent hyperinsulinemia, nutrient excess, and low-grade inflammation are associated with sustained activation of the SREBP pathway, potentially leading to prolonged upregulation of downstream lipogenic enzymes, including *ELOVL6*. Under such conditions, feedback mechanisms that normally constrain SREBP activity may be compromised, resulting in sustained fatty acid remodeling and metabolic stress. A similar alteration in temporal control has been proposed in cancer cells, where oncogenic signaling and metabolic reprogramming may partially uncouple SREBP activity from physiological nutritional cues [12]. Sustained *ELOVL6* expression in malignant cells could contribute to membrane biosynthesis, adaptation to endoplasmic reticulum stress, and proliferative or invasive phenotypes.

Collectively, these considerations suggest that the biological significance of *ELOVL6* regulation may reside not only in its expression level but also in its temporal dynamics. Disruption of transient, pulse-like regulation could represent one step in the transition from adaptive lipid metabolism to chronic metabolic and oncogenic states. These findings may therefore provide a mechanistic framework for understanding how dysregulated *ELOVL6* transcription could contribute to metabolic or oncogenic processes. However, such implications remain speculative and require further experimental validation.

Limitations

This study has several limitations that should be acknowledged. First, the experiments were conducted using a single human hepatoma cell line (Huh7), which may not fully recapitulate the complexity of hepatocyte responses in vivo. Second, the concentration of PA employed, while effective for inducing measurable transcriptional responses under controlled conditions, may exceed physiological levels encountered in certain clinical settings. Third, the present study focused primarily on transcriptional regulation at the promoter level. Functional validation at the level of lipid composition analysis or enzymatic activity was beyond the scope of this investigation and warrants future study. In addition, in vivo validation was not performed. Nuclear SREBP clearance kinetics were inferred from time-course Western blot analysis rather than directly quantified using dedicated protein turnover assays. Direct assessment of degradation dynamics would further refine the mechanistic interpretation of temporal regulatory control. Furthermore, although the present findings support a mechanistic link between fatty acid stimulation and SREBP-mediated regulation of *ELOVL6*, broader implications for metabolic disease or oncogenic contexts should be interpreted cautiously and regarded as hypothesis-generating. Finally, while experiments were performed in three independent biological replicates, the statistical power remains modest, particularly in conditions showing trends without statistical significance. Larger sample sizes and validation across multiple cellular and in vivo models will be necessary to strengthen and extend these observations.

## Conclusions

In this study, we identified functional SREs within a genomic region of the human *ELOVL6* gene encompassing the promoter and first intron and demonstrated that* ELOVL6* transcription is tightly regulated by SREBP in response to fatty acids. Our results indicate that PA induces a transient activation of SREBP signaling, leading to short-term upregulation of *ELOVL6,* whereas this response is attenuated under conditions that disrupt normal feedback control. These findings highlight the importance of temporal regulation of SREBP-*ELOVL6* signaling in maintaining lipid homeostasis and provide a mechanistic framework for understanding how dysregulated fatty acid metabolism contributes to metabolic disease.
